# Prevalence and Causes of Ocular Morbidity in Mbeere District, Kenya. Results of a Population-Based Survey

**DOI:** 10.1371/journal.pone.0070009

**Published:** 2013-08-01

**Authors:** Kahaki Kimani, Robert Lindfield, Laura Senyonjo, Alex Mwaniki, Elena Schmidt

**Affiliations:** 1 Department of Ophthalmology, University of Nairobi, Nairobi, Kenya; 2 International Centre for Eye Health, London School of Hygiene and Tropical Medicine, London, United Kingdom; 3 Sightsavers, Grosvenor Hall, Haywards Health, West Sussex, United Kingdom; 4 Ministry of Planning, Government of Kenya, Nairobi, Kenya; Medical University Graz, Austria

## Abstract

**Purpose:**

Ocular morbidity (OM) describes any eye disease regardless of resultant visual loss. Ocular morbidity may affect large numbers of people in low income countries and could lead to many episodes of care. However there is limited evidence about the prevalence of ocular morbidity or resulting health-seeking behavior. This study in Mbeere District, Kenya, set out to explore both these issues.

**Methods:**

A cross-sectional household survey was conducted in 2011. Trained teams moved from house to house examining and questioning residents on ocular morbidity and health-seeking behavior. Data were collected on standardized proformas and entered into a database for analysis.

**Results:**

3,691 people were examined (response rate 91.7%). 15.52% (95% CI 13.86–16.92) had at least one ocular morbidity in at least one eye. The leading cause was presbyopia which affected 25.11% (95% CI 22.05–28.45) of participants over 35 and increased with age. Other leading causes of OM were conditions that affected the lens (32.58%) and the conjunctiva (31.31%). No association was found between educational attainment or employment and OM. 9.63% (7.87–11.74) self-reported an ocular morbidity in the previous six months and 45.94% (95% CI 37.1–55.04) stated that they had sought treatment for the condition.

**Conclusion:**

A large number of people were affected by an ocular morbidity in this survey. Most of these people could potentially be managed in their own communities through primary care services (e.g. those with presbyopia). Further work is required to understand the best way of providing an effective, equitable service for ocular morbidity.

## Background

Vision2020– The right to Sight, a global initiative to eliminate avoidable blindness, prioritizes blinding eye diseases such as cataract, trachoma, glaucoma, and retinal disease, ninety percent of which occur in low income countries [1]. Whilst this focus on visual impairment has led to strong collaborations and a drive to reduce blindness; other eye conditions, which may affect individuals' functionality and quality of life and might cause people to seek care but do not necessarily lead to blindness, have been overlooked. Consequently the epidemiology and impact of many non-blinding ocular diseases (eg. allergic and infective conjunctivitis, dry eye syndrome, mild refractive error and presbyopia) has not been sufficiently investigated, especially in developing countries, where the availability of evidence is limited by the paucity of population-based studies, unreliable hospital activity data; and extensive use of informal care providers.

The spectrum of eye disease experienced by a population can be termed ‘ocular morbidity’. Ocular morbidity describes eye diseases that are either significant to the individual (the individual is concerned enough about the condition to seek care) or to professionals (an eye health professional determines that the individual would benefit from advice, further review or treatment).

There is limited information about the prevalence of ocular morbidity in populations in developing countries. The few studies that have been conducted suggest that, whilst the majority of eye diseases do not cause visual loss, they may be a significant burden to the population and health system. Thus, in a population-based survey in Nairobi, only 0.1% of the sample was blind, while conjunctival disorders and refractive error (not including presbyopia) were found in 7.6% and 5.6% of the population respectively [2]. A study in Pakistan found prevalence of what the authors termed ‘non-vision impairing conditions’ (NVIC) to be 30.6% including presbyopia. After excluding presbyopia, the prevalence of NVIC was 14.6% with conjunctival disorders (e.g. allergic conjunctivitis) the leading cause [3].

Studies of the impact of NVIC on quality of life are also scarce and confined largely to high income countries; those that are available suggest that these conditions do affect quality of life (QoL) in both general health and specific aspects of vision. A study among patients with seasonal allergic conjunctivitis (SAC) in Spain showed the impact of SAC on the overall vision, distance vision, ocular pain, mental health, role limitations, and dependency [4]. In Singapore symptomatic dry eye was associated with difficulty in performing vision-dependent tasks, such as navigating stairs, recognizing friends, reading road signs and cooking [5]. A few studies conducted in developing countries largely assessed the impact of refractive errors and presbyopia. One study [6] showed that uncorrected presbyopia had a significant impact on the performance of near -vision tasks and vision-related quality of life in rural Tanzania, where over 70% of people with presbyopia were dissatisfied with their ability to do near work. Near vision was needed for a range of tasks, including winnowing and sorting grain, weeding, sewing, cooking, dressing children, and lighting lamps. A study in Zanzibar [7] showed that wearing presbyopic spectacles increased vision function scores, quality of life, patient' satisfaction and willingness to pay for correction.

The lack of knowledge about the prevalence and distribution of various ocular diseases within populations has implications for planning and delivery of eye care services, particularly at primary care level, where many of these conditions could be effectively managed. However, primary care services in low income countries are often insufficiently developed to manage eye diseases. As a result, many individuals with eye complaints, regardless of severity, seek care at more expensive secondary level facilities or do not seek care at all, missing opportunities for early treatment. For example, a study in rural India found that over 58% sought treatment from a general hospital rather than a locally based provider; while two thirds of people with severe visual impairment (eg. cataracts, glaucoma) did not access any healthcare [8].

Without adequate data, advocating for more efficient and responsive eye care services is not possible. There is an urgent need to understand both the burden of ocular morbidity and health-seeking behavior associated with these diseases in resource poor settings. Such information is essential to support decision-making processes, shifting of care and planning of services which meet population needs. The aim of this study was to establish the prevalence and causes of ocular morbidity and to describe eye health seeking behavior in Mbeere District, Kenya.

## Methods

### Study Type

The study was a population-based cross sectional survey. This was selected as it provides robust information about a population (the residents of a specific area) using a representative sample. It was felt that this study type was the most appropriate to explore the research question.

### Survey area

The study was conducted in Mbeere District situated in the Eastern Province of Kenya with an estimated population of 327,262 people [9]. The district is served by two hospitals, three health centers and a number of dispensaries. Mbeere District was chosen as the survey site because it is thought to be representative of the country with respect to demographics, population density, and provision of health and eye care services.

### Sampling

The following were used to calculate the sample size: (a) the prevalence of any OM experienced by the population was estimated to be approximately 10%, (b) precision of 2%; (c) 10% non-response; (d) 95% confidence level; and (d) design effect of 4 (to account for clustering of infective causes of ocular morbidity and cluster design). This gave a sample size of 3500 individuals or 35 clusters of 100 individuals (approximately 20 households per cluster).

A two-stage cluster sampling strategy was used to select the study population. The primary clusters, defined as a village, were randomly selected using probability proportional to size and selected from a sampling frame consisting of all the villages and their sub-locations, as outlined in the 2009 Kenya national census [5]. Compact segment sampling was used to select households within the selected sub-locations. All eligible members of households in the randomly selected segment were enumerated until the cluster sample size (100 individuals) had been obtained. Repeat visits were made the same day or the next day to households (defined as a group of individuals who fulfilled residency requirements (see below) and eat from the same pot) where members were not present. If an eligible member was still missing at the third visit, they were considered as non-responders. Eligibility was defined as staying in the household for at least six of the previous twelve months and sleeping in the house either the previous or the following night.

Non-residents (see definition above) were excluded from the study. Those who refused to take part or were unable to understand the consent process were included as part of the denominator group but no information was collected about them.

Interviews and examinations took place at each household in the selected segments.

### Ethical considerations

The survey was approved by the Kenyatta National Hospital and University of Nairobi Ethical Committee. After explaining the purpose of the study and what was required of a subject, a written informed consent was obtained from head of the household and from each participant or guardian/ parent in the case of a child. All ocular disorders that were treatable in the field were treated and those requiring referral were referred to the local eye unit at Embu District Hospital.

### Data collection

Data were collected by four teams, each consisting of an ophthalmologist, an ophthalmic clinical officer and a community health worker. The principle investigator accompanied different teams throughout the survey period to ensure compliance with the research protocol and consistency in data collection.

The teams received seven days of training prior to the survey. Training took place in a district hospital and the adjacent local area and was led by two experienced ophthalmologists both of whom had previously trained staff for surveys of blindness.

### Defining ocular morbidity

Each team was tasked to determine whether each participant had an ocular morbidity. This meant establishing whether any pathology observed would benefit from advice, further investigation or treatment. Very minor conditions (for example; a pinguecula that did not reach the limbus and was asymptomatic) were excluded. An inter-observer variation test provided a formal assessment of agreement between teams on whether an individual had an ocular morbidity. Teams had to reach a Kappa score of 0.7 before the survey could start.

Three questionnaires were used to collect data, a household questionnaire, an eye examination questionnaire and a health-seeking behavior questionnaire. After written consent from household heads, socio-demographic data and medical histories were recorded for all participants.

All members of the household had their presenting visual acuity measured in each eye using a 6/12 Snellen E optotype for adults and children old enough to respond. Younger children were tested using Lea's Symbols and those under three were assessed using fix and follow.

**Figure 1:**
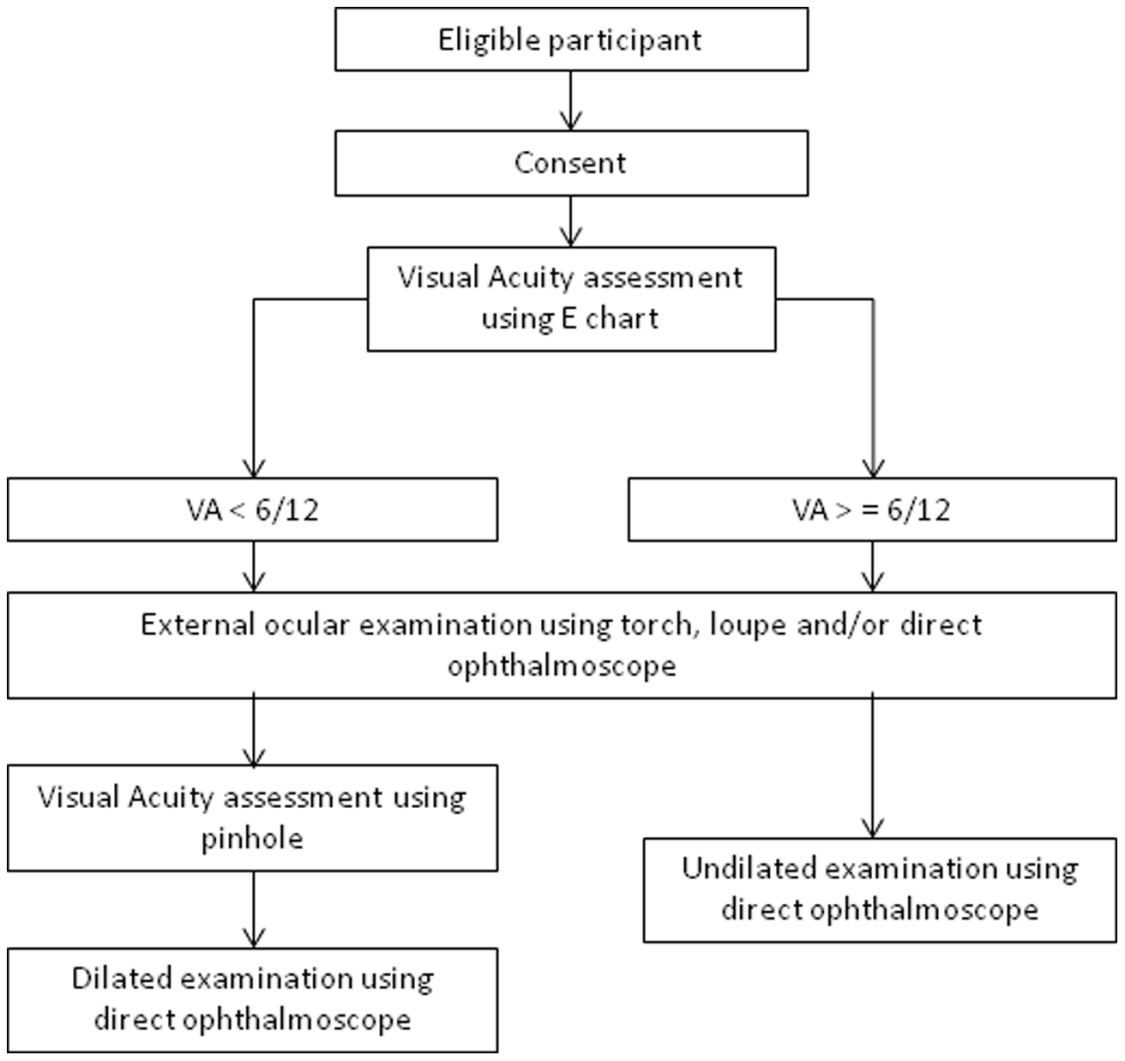
Flow diagram of selection and examination of study participants.

All members of the household underwent a basic eye examination by a trained ophthalmic clinical officer or ophthalmologist using a torch, loupe and direct ophthalmoscope (see Figure 1). All participants with a presenting acuity of worse than 6/12 in one or both eyes were tested with a pinhole and, where possible, also underwent dilated fundoscopy by the ophthalmologist to determine the cause(s) of visual loss. Retinoscopy was carried out in a central location where assessment of best corrected visual acuity was repeated by an optometrist. Intraocular pressure was not measured in the field but participants were defined as a glaucoma suspect if their cup: disc ratio was greater than or equal to 0.8 and they were referred for further assessment.

Individuals aged 35 years or older (determined using a local events calendar or identity card for those who did not know their date of birth) were asked whether they owned spectacles for near activities. All had near acuity tested at 40 cms (with reading glasses for those who had them). The near addition needed to improve their near acuity to at least N8 at 40 cms was recorded.

Each participant was asked whether they had experienced any problem with their eyes in the last six months. Those who had reported problems were interviewed by the community health worker using the health seeking behavior questionnaire.

### Data management and analysis

All completed questionnaires were collected on a daily basis and checked for quality of completion by team leaders.

The data were entered to the Microsoft Access database using double entry on a daily basis and validated using MS Excel. Analysis was performed using STATA 12.0.

Descriptive statistics were employed to present simple frequencies of the dependent variable and their distribution by sex, age, education and occupation. Chi-squared tests, logistic regression and multivariate logistic regression models, adjusted for clustering, were used to assess for risk factors associated with an ocular morbidity.

## Results

### Study participants

A total of 938 households participated in the survey, with 4,023 persons listed as normally resident in these households and eligible to take part in the survey. A total of 3,691 persons were examined for signs of an ocular morbidity, giving a response rate of 91.7%. Amongst those that were examined, 56.90% (95% CI 55.24–58.54) were female; the median age of participants was 17 years. Of those aged over 15, the majority (61.37% (95% CI 58.06–64.58)) had only attended primary school, and 15.08% (12.68–17.83) had no education. The majority of adults (64.31% (95% CI 60.09–68.33) were involved in some form of manual labor.

### Prevalence of Ocular Morbidity

563 individuals or 15.52% (95% CI 13.86–16.92) of those examined had at least one type of ocular morbidity (including presbyopia or refractive error) in at least one eye. A total of 1.88% (1.41–2.50) of the study population had more than one ocular morbidity at the time of the survey. Table 1 shows the breakdown of any ocular morbidity by gender, age, education and occupation.

**Table 1:** Prevalence of at least one ocular morbidity in at least one eye by gender, age, education and occupation.

Characteristic	Total n	% OM (n)	95%CI	Univariate OR (95% CI)	p value
**Age (years)**					
0–4	591	6.26 (37)	4.63–8.42	1	
5–14	1,105	6.34 (70)	4.74–8.42	1.01 (0.68–1.52)	0.95
15–34	932	6.01 (56)	4.67–7.71	0.96 (0.63–1.46)	0.84
35–54	566	23.85 (135)	20.15–28.0	4.69 (3.14–7.01)	<0.001
55–74	324	54.01 (175)	48.63–59.31	17.59 (11.63–26.59)	<0.001
75+	109	82.57 (90)	74.47–88.50	70.93 (40.67–123.67)	<0.001
**Sex**					
Male	1,550	14.71 (228)	12.79–16.86	1	
Female	2,077	16.13 (335)	14.34–18.1	1.12 (0.93–1.34)	0.24
**Education (if over 15)**					
No schooling	289	53.98 (156)	48.72–59.15	1	
Primary	1,184	18.58 (220)	15.92–21.57	0.19 (0.15–0.26)	<0.001
Secondary	382	16.75 (64)	13.28–20.92	0.17 (0.12–0.24)	<0.001
Tertiary	76	21.05 (16)	11.46–35.47	0.23 (0.10–0.49)	<0.001
**Occupation (if over 15)**					
Non-manual work	476	18.10 (38)	14.51–22.33	1	
Not working (retired, student, housewife)	210	20.38 (97)	17.06–24.16	1.15 (0.82–1.63)	0.39
Manual work	1245	25.78 (321)	22.8–29.01	1.57 (1.15–2.15)	0.006

The prevalence of ocular morbidity increased with age (p<0.001), ranging from 6.26% in children under five, increasing to 82.57% in adults 75 or over. We found no association between ocular morbidity and sex but the univariate odds ratios (Table 1) suggest that people with no education and those in manual work were more likely to have an ocular morbidity. After adjusting for age, these associations disappeared.

### Causes of Ocular Morbidity

For the purposes of this study, presbyopia and refractive error (defined as not being able to see 6/12 unaided but able to see 6/12 with pinhole or best correction) were included in the definition of ocular morbidity. Presbyopia (in adults aged 35 or older) was a significant cause of an ocular morbidity, with 226 people or 25.11% (22.05–28.45) of those sampled unable to see N8 at 40 cms without correction. Only 9.96% (6.43–15.11) of those identified with presbyopia, reported having reading glasses. As expected, presbyopia increased with age (Table 2).

**Table 2:** Prevalence of presbyopia by age.

Age group	%	95%CI	n
35–54	13.33	10.31–17.08	68
55–74	36.08	31.1–41.38	105
75+	53.54	43.19–63.58	53
Total	25.11	22.05–28.45	226

5.18% (4.32–6.21) of over fives could not see 6/12 in the better eye, there was no evidence of any difference between sexes (p = 0.76). Of those over 5 years old who could not see 6/12, 36.91% (26.07–49.25) could see 6/12 with pinhole. In children under five, 0.89% (0.19–4.15) could not fix and follow, whilst all children between ages four and five could see 6/12. In the whole population 1.1% of people wore distance glasses and 1.6% reading glasses.

Further information was obtained on the anatomical site of the ocular morbidity. Amongst those that had an ocular morbidity (excluding persons with presbyopia or refractive error), the majority were conditions that affected the lens (32.58%) and the conjunctiva (31.31%) (Table 3). Other conditions were less prevalent including those affecting the cornea (12.63%), optic nerve (9.60%) and retina (7.07%).

**Table 3:** Anatomical site of ocular morbidity.

Site of OM	% of persons with an ocular morbidity in the site	95% CI
Orbit and globe	6.31	4.31–9.15
Eyelid	9.09	6.14–13.26
Conjunctiva	31.31	24.30–39.30
Cornea	12.63	9.01–17.41
Pupil	7.32	5.08–10.55
Lens	32.58	27.68–37.89
Optic nerve	9.60	6.69–13.59
Retina	7.07	4.79–10.32

There was strong evidence that the prevalence of an ocular morbidity affecting the lens increased with age (p<0.001), while, the prevalence of an ocular morbidity of the conjunctiva decreased with age (p<0.001).

### Treatment seeking behavior

58.89% (53.75–63.83) of those with an identified ocular morbidity on the day of the survey were referred for further management or provided with some form of treatment (topical or spectacles) by the survey teams.

A total of 9.63% (7.87–11.74) of those surveyed self-reported that they had had an ocular morbidity in the six months prior to the survey. Of those 45.94% (37.1–55.04) stated that they had sought treatment, mainly from a health worker, doctor or optician (81.63%, 71.49–88.74). No individual reported seeking care from a traditional healer.

## Discussion

This survey found that nearly one in six individuals in Mbeere District in Kenya had at least one ocular morbidity in at least one eye at the time of the survey. The results are similar to the findings from other studies in Kenya [2], suggesting that ocular morbidity, including blinding and non-blinding conditions, is a significant public health issue which requires an adequate health system response.

If the total prevalence estimate of OM in this study is crudely applied to the total population then over 50,000 people in Mbeere District would have an ocular morbidity at any point in time; and the majority of them would need some form of treatment. Our data on health seeking behavior in this study were based on self-reporting and needs to be treated with caution; but if correct it indicates significant unmet need for eye care in this community. Thus, while nearly 60% of our participants with a diagnosed ocular morbidity required medical care at the time of the study, only 45% of those who self-reported eye problems in the past six months sought some form of advice outside their home and we do not know about the effectiveness or quality of services they received. Our findings do not support the common assumption that most ocular morbidity is self-limiting and does not require assessment; the scale of ocular morbidity and potential demand for services needs to be taken into account when planning eye care services at a district level.

Similarly to other studies of OM [2], [3], [10], [11], the diseases affecting the lens and conjunctiva were among the most common eye conditions. There was a strong association between these morbidities and age: Lens conditions affected older age groups, most likely reflecting the higher prevalence of cataract amongst people over 50, conjunctival conditions were associated with younger age, most likely due to high prevalence of allergic conjunctivitis amongst younger people. Age and characteristics of the affected populations need to be taken into account in healthcare planning, as they are the likely determinants of health seeking behavior and uptake of services.

A quarter of those over 35 had presbyopia. Although most participants did not complain of poor near vision (possibly reflecting the fact that their occupation or day-to-day activity did not require much near work), the findings suggest that there is a significant unmet need for reading glasses in this population, as only one in ten of those who needed reading glasses had them. The high prevalence of presbyopia amongst older population groups in sub-Saharan Africa has been reported in many studies [11]–[14]. Standard magnifying spectacles have become increasingly available in shops throughout sub-Saharan Africa which suggests that shifting care away from more expensive secondary services and managing the condition at community or primary care level may be possible and appropriate.

A number of limitations need to be taken into account when considering results of this study.

The main limitation was diagnostic uncertainty. This resulted from teams using only torches and direct ophthalmoscopes to examine participants. Consequently no detailed diagnoses were possible and results were grouped based on the anatomical sites they affected.

Diagnostic categorization was tested using a formal inter-observer variation test in a hospital setting. It is possible that in a community setting different disease entities or severities were encountered. This may have led to variation in diagnoses given by different teams, although reports from the ophthalmologists working on the survey suggested that this was not the case and that the spectrum of disease in the community was similar to that in the clinic.

It is also not possible to predict the impact on clinical services of this prevalence of ocular morbidity. Approximately 50% of individuals with an ocular morbidity were treated or referred by the survey teams suggesting that the other 50% had mild disease that the ophthalmologist did not feel required treatment. This survey did not determine whether those with ‘mild’ disease felt, in the absence of the survey, that they would seek advice about their condition potentially underestimating the numbers who would seek services.

In conclusion, the findings from Mbeere District, Kenya suggest that a range of different eye diseases affect a large number of individuals at any one time. Some of these conditions require medical or surgical attention, others potentially can be managed in local health facilities and some are self-limiting. Providing services for these people requires a range of different approaches at community, primary and secondary eye care levels. There is an urgent need to have evidence-based policy discussions regarding a range of services, which can be adequately delivered at each level of care; and about the capacity of the system required. Particular attention needs to be given to essential equipment and supplies, training and supervision of staff and adequate information and referral systems. Shifting of certain eye care services from secondary to community and primary care may bring efficiency gains and improve access to services, providing these services are appropriately planned and have the capacity to deliver effective and safe care, which local communities are willing to use.
